# Supplementary material for: COVID-19 and human-nature relationships: Vermonters’ activities in nature and associated nonmaterial values during the pandemic
Source: PLoS One. 2020 Dec 11;15(12):e0243697. doi: 10.1371/journal.pone.0243697 (PMC7732125; doi:10.1371/journal.pone.0243697)
Supplement: S1 Metadata — (PDF) [file pone.0243697.s002.pdf]

COVID-19 and Human-Nature Relationships

Title	COVID-19 and Human-Nature Relationships
Subtitle	Vermonters’ Activities in Nature and Associated Nonmaterial Values during the Pandemic
Creator	Josh Morse
Contributor	Tatiana Gladkikh, Diana Hackenburg, Rachelle Gould
Variable Count	127

ID

Type	Numeric (Integer)
Numeric Details	Decimals: 0
Description	Raw unique numeric identifier for each survey respondent
Analysis Unit	Individual
Response Unit	Informant

ActivityEquation

Type	Code
Description	Level of participation by activity in this order: Biking; Boating; Camping; Fishing; Gardening and yardwork; Gathering wild food (plants, mushrooms, etc.); Hiking; Hunting; Jogging or running; Photography, drawing, or other art; Relaxing with friends or family in nature; Relaxing by myself in nature; rock climbing; walking; watching wildlife
Analysis Unit	Individual
Response Unit	Informant

A1	I don't do this activity
A2	Much less
A3	Less
A4	Somewhat less
A5	Same amount
A6	Somewhat more
A7	More
A8	Much more

A1	I don't do this activity

A2	Much less
A3	Less
A4	Somewhat less
A5	Same amount
A6	Somewhat more
A7	More
A8	Much more

BikingOrdinalScaleNum

Type	Code
Description	Condensed numeric variable for levels of activity engagement derived from the raw “Biking" column
Analysis Unit	Individual
Response Unit	Informant

0	Did not engage
1	Less engagement
2	No change
3	More engagement

0	Did not engage
1	Less engagement
2	No change
3	More engagement

BikingOrdinalScaleCat

Type	Code
Description	Condensed categorical variable for levels of activity engagement derived from the raw “Biking” column
Analysis Unit	Individual
Response Unit	Informant

Doesn't do activity	A1
Less	A2, A3, or A4

More	A6, A7, or A8
No change	A5

Doesn't do activity	A1
Less	A2, A3, or A4
More	A6, A7, or A8
No change	A5

Biking

Type	Code
Description	Raw responses for Biking engagement for the question: "Compared to this time last year, how frequently are you participating in these activities in a typical month?"
Analysis Unit	Individual
Response Unit	Informant

A1	I don't do this activity
A2	Much less
A3	Less
A4	Somewhat less
A5	Same amount
A6	Somewhat more
A7	More
A8	Much more

A1	I don't do this activity
A2	Much less
A3	Less
A4	Somewhat less
A5	Same amount
A6	Somewhat more
A7	More
A8	Much more

BoatingOrdinalScaleNum

Type	Code
Description	Condensed numeric variable for levels of activity engagement derived from the raw “Boating" column
Analysis Unit	Individual
Response Unit	Informant

0	Did not engage
1	Less engagement
2	No change
3	More engagement

0	Did not engage
1	Less engagement
2	No change
3	More engagement

BoatingOrdinalScaleCat

Type	Code
Description	Condensed categorical variable for levels of activity engagement derived from the raw “Boating" column
Analysis Unit	Individual
Response Unit	Informant

Doesn't do activity	A1
Less	A2, A3, or A4
More	A6, A7, or A8
No change	A5

Doesn't do activity	A1
Less	A2, A3, or A4
More	A6, A7, or A8
No change	A5

Boating

Type	Code
Description	Raw responses for Boating or paddling engagement for the question: "Compared to this time last year, how frequently are you participating in these activities in a typical month?"
Analysis Unit	Individual
Response Unit	Informant

A1	I don't do this activity
A2	Much less
A3	Less
A4	Somewhat less
A5	Same amount
A6	Somewhat more
A7	More
A8	Much more

A1	I don't do this activity
A2	Much less
A3	Less
A4	Somewhat less
A5	Same amount
A6	Somewhat more
A7	More
A8	Much more

CampingOrdinalScaleNum

Type	Code
Description	Condensed numeric variable for levels of activity engagement derived from the raw “Camping" column
Analysis Unit	Individual
Response Unit	Informant

0	Did not engage

1	Less engagement
2	No change
3	More engagement

0	Did not engage
1	Less engagement
2	No change
3	More engagement

CampingOrdinalScaleCat

Type	Code
Description	Condensed categorical variable for levels of activity engagement derived from the raw “Biking” column
Analysis Unit	Individual
Response Unit	Informant

Doesn't do activity	A1
Less	A2, A3, or A4
More	A6, A7, or A8
No change	A5

Doesn't do activity	A1
Less	A2, A3, or A4
More	A6, A7, or A8
No change	A5

Camping

Type	Code
Description	Raw responses for Camping engagement for the question: "Compared to this time last year, how frequently are you participating in these activities in a typical month?"
Analysis Unit	Individual
Response Unit	Informant

A1	I don't do this activity

A2	Much less
A3	Less
A4	Somewhat less
A5	Same amount
A6	Somewhat more
A7	More
A8	Much more

A1	I don't do this activity
A2	Much less
A3	Less
A4	Somewhat less
A5	Same amount
A6	Somewhat more
A7	More
A8	Much more

FishingOrdinalScaleNum

Type	Code
Description	Condensed numeric variable for levels of activity engagement derived from the raw “Fishing" column
Analysis Unit	Individual
Response Unit	Informant

0	Did not engage
1	Less engagement
2	No change
3	More engagement

0	Did not engage
1	Less engagement
2	No change
3	More engagement

FishingOrdinalScaleCat

Type	Code
Description	Condensed categorical variable for levels of activity engagement derived from the raw “Fishing” column
Analysis Unit	Individual
Response Unit	Informant

Doesn't do activity	A1
Less	A2, A3, or A4
More	A6, A7, or A8
No change	A5

Doesn't do activity	A1
Less	A2, A3, or A4
More	A6, A7, or A8
No change	A5

Fishing

Type	Code
Description	Raw responses for Fishing engagement for the question: "Compared to this time last year, how frequently are you participating in these activities in a typical month?"
Analysis Unit	Individual
Response Unit	Informant

A1	I don't do this activity
A2	Much less
A3	Less
A4	Somewhat less
A5	Same amount
A6	Somewhat more
A7	More
A8	Much more

A1	I don't do this activity
A2	Much less
A3	Less
A4	Somewhat less
A5	Same amount
A6	Somewhat more
A7	More
A8	Much more

GardeningOrdinalScaleNum

Type	Code
Description	Condensed numeric variable for levels of activity engagement derived from the raw “Gardening" column
Analysis Unit	Individual
Response Unit	Informant

0	Did not engage
1	Less engagement
2	No change
3	More engagement

0	Did not engage
1	Less engagement
2	No change
3	More engagement

GardeningOrdinalScaleCat

Type	Code
Description	Condensed categorical variable for levels of activity engagement derived from the raw “Gardening” column
Analysis Unit	Individual
Response Unit	Informant

Doesn't do activity	A1

Less	A2, A3, or A4
More	A6, A7, or A8
No change	A5

Doesn't do activity	A1
Less	A2, A3, or A4
More	A6, A7, or A8
No change	A5

Gardening

Type	Code
Description	Raw responses for Gardening or yardwork engagement for the question: "Compared to this time last year, how frequently are you participating in these activities in a typical month?"
Analysis Unit	Individual
Response Unit	Informant

A1	I don't do this activity
A2	Much less
A3	Less
A4	Somewhat less
A5	Same amount
A6	Somewhat more
A7	More
A8	Much more

A1	I don't do this activity
A2	Much less
A3	Less
A4	Somewhat less
A5	Same amount
A6	Somewhat more
A7	More

A8	Much more

GatheringOrdinalScaleNum

Type	Code
Description	Condensed numeric variable for levels of activity engagement derived from the raw “Gathering" column
Analysis Unit	Individual
Response Unit	Informant

0	Did not engage
1	Less engagement
2	No change
3	More engagement

0	Did not engage
1	Less engagement
2	No change
3	More engagement

GatheringOrdinalScaleCat

Type	Code
Description	Condensed categorical variable for levels of activity engagement derived from the raw “Gathering” column
Analysis Unit	Individual
Response Unit	Informant

Doesn't do activity	A1
Less	A2, A3, or A4
More	A6, A7, or A8
No change	A5

Doesn't do activity	A1
Less	A2, A3, or A4
More	A6, A7, or A8

No change	A5

Gathering

Type	Code
Description	Raw responses for Gathering wild food (plants, mushrooms, etc.) engagement for the question: "Compared to this time last year, how frequently are you participating in these activities in a typical month?"
Analysis Unit	Individual
Response Unit	Informant

A1	I don't do this activity
A2	Much less
A3	Less
A4	Somewhat less
A5	Same amount
A6	Somewhat more
A7	More
A8	Much more

A1	I don't do this activity
A2	Much less
A3	Less
A4	Somewhat less
A5	Same amount
A6	Somewhat more
A7	More
A8	Much more

HikingOrdinalScaleNum

Type	Code
Description	Condensed numeric variable for levels of activity engagement derived from the raw “Hiking" column
Analysis Unit	Individual
Response Unit	Informant

0	Did not engage
1	Less engagement
2	No change
3	More engagement

0	Did not engage
1	Less engagement
2	No change
3	More engagement

HikingOrdinalScaleCat

Type	Code
Description	Condensed categorical variable for levels of activity engagement derived from the raw “Hiking” column
Analysis Unit	Individual
Response Unit	Informant

Doesn't do activity	A1
Less	A2, A3, or A4
More	A6, A7, or A8
No change	A5

Doesn't do activity	A1
Less	A2, A3, or A4
More	A6, A7, or A8
No change	A5

Hiking

Type	Code
Description	Raw responses for Hiking engagement for the question: "Compared to this time last year, how frequently are you participating in these activities in a typical month?"
Analysis Unit	Individual
Response Unit	Informant

A1	I don't do this activity
A2	Much less
A3	Less
A4	Somewhat less
A5	Same amount
A6	Somewhat more
A7	More
A8	Much more

A1	I don't do this activity
A2	Much less
A3	Less
A4	Somewhat less
A5	Same amount
A6	Somewhat more
A7	More
A8	Much more

HuntingOrdinalScaleNum

Type	Code
Description	Condensed numeric variable for levels of activity engagement derived from the raw “Hunting" column
Analysis Unit	Individual
Response Unit	Informant

0	Did not engage
1	Less engagement
2	No change
3	More engagement

0	Did not engage
1	Less engagement
2	No change
3	More engagement

HuntingOrdinalScaleCat

Type	Code
Description	Condensed categorical variable for levels of activity engagement derived from the raw “Hunting” column
Analysis Unit	Individual
Response Unit	Informant

Doesn't do activity	A1
Less	A2, A3, or A4
More	A6, A7, or A8
No change	A5

Doesn't do activity	A1
Less	A2, A3, or A4
More	A6, A7, or A8
No change	A5

Hunting

Type	Code
Description	Raw responses for Hunting engagement for the question: "Compared to this time last year, how frequently are you participating in these activities in a typical month?"
Analysis Unit	Individual
Response Unit	Informant

A1	I don't do this activity
A2	Much less
A3	Less
A4	Somewhat less
A5	Same amount
A6	Somewhat more
A7	More
A8	Much more

A1	I don't do this activity
A2	Much less
A3	Less
A4	Somewhat less
A5	Same amount
A6	Somewhat more
A7	More
A8	Much more

JoggingOrdinalScaleNum

Type	Code
Description	Condensed numeric variable for levels of activity engagement derived from the raw “Jogging" column
Analysis Unit	Individual
Response Unit	Informant

0	Did not engage
1	Less engagement
2	No change
3	More engagement

0	Did not engage
1	Less engagement
2	No change
3	More engagement

JoggingOrdinalScaleCat

Type	Code
Description	Condensed categorical variable for levels of activity engagement derived from the raw “Jogging” column
Analysis Unit	Individual
Response Unit	Informant

Doesn't do activity	A1

Less	A2, A3, or A4
More	A6, A7, or A8
No change	A5

Doesn't do activity	A1
Less	A2, A3, or A4
More	A6, A7, or A8
No change	A5

Jogging

Type	Code
Description	Raw responses for Jogging or running engagement for the question: "Compared to this time last year, how frequently are you participating in these activities in a typical month?"
Analysis Unit	Individual
Response Unit	Informant

A1	I don't do this activity
A2	Much less
A3	Less
A4	Somewhat less
A5	Same amount
A6	Somewhat more
A7	More
A8	Much more

A1	I don't do this activity
A2	Much less
A3	Less
A4	Somewhat less
A5	Same amount
A6	Somewhat more
A7	More

A8	Much more

PhotographyOrdinalScaleNum

Type	Code
Description	Condensed numeric variable for levels of activity engagement derived from the raw “Photography" column
Analysis Unit	Individual
Response Unit	Informant

0	Did not engage
1	Less engagement
2	No change
3	More engagement

0	Did not engage
1	Less engagement
2	No change
3	More engagement

PhotographyOrdinalScaleCat

Type	Code
Description	Condensed categorical variable for levels of activity engagement derived from the raw “Photography” column
Analysis Unit	Individual
Response Unit	Informant

Doesn't do activity	A1
Less	A2, A3, or A4
More	A6, A7, or A8
No change	A5

Doesn't do activity	A1
Less	A2, A3, or A4
More	A6, A7, or A8

No change	A5

Photography

Type	Code
Description	Raw responses for Photography, drawing, or other art engagement for the question: "Compared to this time last year, how frequently are you participating in these activities in a typical month?"
Analysis Unit	Individual
Response Unit	Informant

A1	I don't do this activity
A2	Much less
A3	Less
A4	Somewhat less
A5	Same amount
A6	Somewhat more
A7	More
A8	Much more

A1	I don't do this activity
A2	Much less
A3	Less
A4	Somewhat less
A5	Same amount
A6	Somewhat more
A7	More
A8	Much more

RelaxOthersOrdinalScaleNum

Type	Code
Description	Condensed numeric variable for levels of activity engagement derived from the raw “RelaxOthers" column
Analysis Unit	Individual
Response Unit	Informant

0	Did not engage
1	Less engagement
2	No change
3	More engagement

0	Did not engage
1	Less engagement
2	No change
3	More engagement

RelaxOthersOrdinalScaleCat

Type	Code
Description	Condensed categorical variable for levels of activity engagement derived from the raw “RelaxOthers” column
Analysis Unit	Individual
Response Unit	Informant

Doesn't do activity	A1
Less	A2, A3, or A4
More	A6, A7, or A8
No change	A5

Doesn't do activity	A1
Less	A2, A3, or A4
More	A6, A7, or A8
No change	A5

RelaxOthers

Type	Code
Description	Raw responses for Relaxing with friends or family in nature engagement for the question: "Compared to this time last year, how frequently are you participating in these activities in a typical month?"
Analysis Unit	Individual
Response Unit	Informant

A1	I don't do this activity
A2	Much less
A3	Less
A4	Somewhat less
A5	Same amount
A6	Somewhat more
A7	More
A8	Much more

A1	I don't do this activity
A2	Much less
A3	Less
A4	Somewhat less
A5	Same amount
A6	Somewhat more
A7	More
A8	Much more

RelaxMyselfOrdinalScaleNum

Type	Code
Description	Condensed numeric variable for levels of activity engagement derived from the raw “RelaxMyself" column
Analysis Unit	Individual
Response Unit	Informant

0	Did not engage
1	Less engagement
2	No change
3	More engagement

0	Did not engage
1	Less engagement
2	No change
3	More engagement

RelaxMyselfOrdinalScaleCat

Type	Code
Description	Condensed categorical variable for levels of activity engagement derived from the raw “RelaxMyself” column
Analysis Unit	Individual
Response Unit	Informant

Doesn't do activity	A1
Less	A2, A3, or A4
More	A6, A7, or A8
No change	A5

Doesn't do activity	A1
Less	A2, A3, or A4
More	A6, A7, or A8
No change	A5

RelaxMyself

Type	Code
Description	Raw responses for Relaxing by myself in nature engagement for the question: "Compared to this time last year, how frequently are you participating in these activities in a typical month?"
Analysis Unit	Individual
Response Unit	Informant

A1	I don't do this activity
A2	Much less
A3	Less
A4	Somewhat less
A5	Same amount
A6	Somewhat more
A7	More
A8	Much more

A1	I don't do this activity
A2	Much less
A3	Less
A4	Somewhat less
A5	Same amount
A6	Somewhat more
A7	More
A8	Much more

RockClimbingOrdinalScaleNum

Type	Code
Description	Condensed numeric variable for levels of activity engagement derived from the raw “RockClimbing" column
Analysis Unit	Individual
Response Unit	Informant

0	Did not engage
1	Less engagement
2	No change
3	More engagement

0	Did not engage
1	Less engagement
2	No change
3	More engagement

RockClimbingOrdinalScaleCat

Type	Code
Description	Condensed categorical variable for levels of activity engagement derived from the raw “RockClimbing” column
Analysis Unit	Individual
Response Unit	Informant

Doesn't do activity	A1

Less	A2, A3, or A4
More	A6, A7, or A8
No change	A5

Doesn't do activity	A1
Less	A2, A3, or A4
More	A6, A7, or A8
No change	A5

RockClimbing

Type	Code
Description	Raw responses for Rock Climbing engagement for the question: "Compared to this time last year, how frequently are you participating in these activities in a typical month?"
Analysis Unit	Individual
Response Unit	Informant

A1	I don't do this activity
A2	Much less
A3	Less
A4	Somewhat less
A5	Same amount
A6	Somewhat more
A7	More
A8	Much more

A1	I don't do this activity
A2	Much less
A3	Less
A4	Somewhat less
A5	Same amount
A6	Somewhat more
A7	More

A8	Much more

WalkingOrdinalScaleNum

Type	Code
Description	Condensed numeric variable for levels of activity engagement derived from the raw “Walking" column
Analysis Unit	Individual
Response Unit	Informant

0	Did not engage
1	Less engagement
2	No change
3	More engagement

0	Did not engage
1	Less engagement
2	No change
3	More engagement

WalkingOrdinalScaleCat

Type	Code
Description	Condensed categorical variable for levels of activity engagement derived from the raw “Walking” column
Analysis Unit	Individual
Response Unit	Informant

Doesn't do activity	A1
Less	A2, A3, or A4
More	A6, A7, or A8
No change	A5

Doesn't do activity	A1
Less	A2, A3, or A4
More	A6, A7, or A8

No change	A5

Walking

Type	Code
Description	Raw responses for Walking engagement for the question: "Compared to this time last year, how frequently are you participating in these activities in a typical month?"
Analysis Unit	Individual
Response Unit	Informant

A1	I don't do this activity
A2	Much less
A3	Less
A4	Somewhat less
A5	Same amount
A6	Somewhat more
A7	More
A8	Much more

A1	I don't do this activity
A2	Much less
A3	Less
A4	Somewhat less
A5	Same amount
A6	Somewhat more
A7	More
A8	Much more

WildlifeOrdinalScaleNum

Type	Code
Description	Condensed numeric variable for levels of activity engagement derived from the raw “Wildlife" column
Analysis Unit	Individual
Response Unit	Informant

0	Did not engage
1	Less engagement
2	No change
3	More engagement

0	Did not engage
1	Less engagement
2	No change
3	More engagement

WildlifeOrdinalScaleCat

Type	Code
Description	Condensed categorical variable for levels of activity engagement derived from the raw “Wildlife” column
Analysis Unit	Individual
Response Unit	Informant

Doesn't do activity	A1
Less	A2, A3, or A4
More	A6, A7, or A8
No change	A5

Doesn't do activity	A1
Less	A2, A3, or A4
More	A6, A7, or A8
No change	A5

Wildlife - Watching Wildlife

Type	Code
Description	Raw responses for Watching Wildlife engagement for the question: "Compared to this time last year, how frequently are you participating in these activities in a typical month?"
Analysis Unit	Individual
Response Unit	Informant

A1	I don't do this activity
A2	Much less
A3	Less
A4	Somewhat less
A5	Same amount
A6	Somewhat more
A7	More
A8	Much more

A1	I don't do this activity
A2	Much less
A3	Less
A4	Somewhat less
A5	Same amount
A6	Somewhat more
A7	More
A8	Much more

BENE_AESTH - Benefit Aesthetics

Type	Numeric (Integer)
Measurement Unit	Strongly disagree (1) to Strongly agree (7)
Numeric Details	Decimals: 0
Description	Raw responses for participants’ agreement with "I appreciate nature’s sights, smells, sounds, etc."
Analysis Unit	Individual
Response Unit	Informant

BENE_CREATIVE - Benefit Creativity

Type	Numeric (Integer)
Measurement Unit	Strongly disagree (1) to Strongly agree (7)
Numeric Details	Decimals: 0
Description	Raw responses for participants’ agreement with "Nature inspires me to be creative and express myself"

Analysis Unit	Individual
Response Unit	Informant

BENE_TRADITION - Benefit Tradition

Type	Numeric (Integer)
Measurement Unit	Strongly disagree (1) to Strongly agree (7)
Description	Raw responses for participants’ agreement with "Through experiences of nature, I connect with traditions that are important to me"
Analysis Unit	Individual
Response Unit	Informant

BENE_FOOD - Benefit Food

Type	Numeric (Integer)
Measurement Unit	Strongly disagree (1) to Strongly agree (7)
Numeric Details	Decimals: 0
Description	Raw responses for participants’ agreement with "I rely on nature—a garden, foraging spot, hunting place, etc.—for food"
Analysis Unit	Individual
Response Unit	Informant

BENE_ID - Benefit Identity

Type	Numeric (Integer)
Measurement Unit	Strongly disagree (1) to Strongly agree (7)
Numeric Details	Decimals: 0
Description	Raw responses for participants’ agreement with "I consider experiences of nature as important to my sense of who I am"
Analysis Unit	Individual
Response Unit	Informant

BENE_LIFELESSON - Benefit Life Lesson

Type	Numeric (Integer)
Measurement Unit	Strongly disagree (1) to Strongly agree (7)
Numeric Details	Decimals: 0
Description	Raw responses for participants’ agreement with "I learn important lessons about life from my contact with nature"

Analysis Unit	Individual
Response Unit	Informant

BENE_MENTALWB - Benefit Mental Well-being

Type	Numeric (Integer)
Measurement Unit	Strongly disagree (1) to Strongly agree (7)
Numeric Details	Decimals: 0
Description	Raw responses for participants’ agreement with "Experiences of nature contribute to my mental well-being"
Analysis Unit	Individual
Response Unit	Informant

BENE_EXER - Benefit Exercise

Type	Numeric (Integer)
Measurement Unit	Strongly disagree (1) to Strongly agree (7)
Numeric Details	Decimals: 0
Description	Raw responses for participants’ agreement with "Time in nature helps me stay in shape and get my exercise"
Analysis Unit	Individual
Response Unit	Informant

BENE_FUN - Benefit Fun

Type	Numeric (Integer)
Measurement Unit	Strongly disagree (1) to Strongly agree (7)
Numeric Details	Decimals: 0
Description	Raw responses for participants’ agreement with "I spend time in nature for leisure and fun"
Analysis Unit	Individual
Response Unit	Informant

BENE_FAMILIAR - Benefit Familiar

Type	Numeric (Integer)
Measurement Unit	Strongly disagree (1) to Strongly agree (7)
Numeric Details	Decimals: 0

Description	Raw responses for participants’ agreement with "I appreciate the feeling of familiarity I have with places in nature that I know well"
Analysis Unit	Individual
Response Unit	Informant

BENE_SOCIAL - Benefit Social

Type	Numeric (Integer)
Measurement Unit	Strongly disagree (1) to Strongly agree (7)
Numeric Details	Decimals: 0
Description	Raw responses for participants’ agreement with "Experiences in nature are an important part of my social life"
Analysis Unit	Individual
Response Unit	Informant

BENE_SPIRTUAL - Benefit Spiritual

Type	Numeric (Integer)
Measurement Unit	Strongly disagree (1) to Strongly agree (7)
Numeric Details	Decimals: 0
Description	Raw responses for participants’ agreement with "I feel a connection to something bigger than myself through experiences in nature"
Analysis Unit	Individual
Response Unit	Informant

BENE_CARE - Benefit Care

Type	Numeric (Integer)
Measurement Unit	Strongly disagree (1) to Strongly agree (7)
Numeric Details	Decimals: 0
Description	Raw responses for participants’ agreement with "I find meaning by caring for nature"
Analysis Unit	Individual
Response Unit	Informant

BENE_R_AESTH - Benefit Ranking Aesthetic

Type	Code
Description	Based on variables in columns BenefitsRank[1], BenefitsRank[2], and BenefitsRank[3]

Analysis Unit	Individual
Response Unit	Informant

Benefit Ranking - Aesthetic

0	Not ranked
1	BenefitsRank [1] - Most important
2	BenefitsRank[2] - Second most important
3	BenefitsRank[3] - Third most important
99	Did not rank values

0	Not ranked
1	BenefitsRank [1] - Most important
2	BenefitsRank[2] - Second most important
3	BenefitsRank[3] - Third most important
99	Did not rank values

BENE_R_CREATIVE - Benefit Ranking Creativity

Type	Code
Description	Based on variables in columns BenefitsRank[1], BenefitsRank[2], and BenefitsRank[3]

Benefit Ranking - Aesthetic

0	Not ranked
1	BenefitsRank [1] - Most important
2	BenefitsRank[2] - Second most important
3	BenefitsRank[3] - Third most important
99	Did not rank values

0	Not ranked
1	BenefitsRank [1] - Most important
2	BenefitsRank[2] - Second most important
3	BenefitsRank[3] - Third most important
99	Did not rank values

BENE_R_TRADITION - Benefit Ranking Tradition

Type	Code
Description	Based on variables in columns BenefitsRank[1], BenefitsRank[2], and BenefitsRank[3]

Benefit Ranking - Aesthetic

0	Not ranked
1	BenefitsRank [1] - Most important
2	BenefitsRank[2] - Second most important
3	BenefitsRank[3] - Third most important
99	Did not rank values

0	Not ranked
1	BenefitsRank [1] - Most important
2	BenefitsRank[2] - Second most important
3	BenefitsRank[3] - Third most important
99	Did not rank values

BENE_R_FOOD - Benefit Ranking Food

Type	Code
Description	Based on variables in columns BenefitsRank[1], BenefitsRank[2], and BenefitsRank[3]

Benefit Ranking - Aesthetic

0	Not ranked
1	BenefitsRank [1] - Most important
2	BenefitsRank[2] - Second most important
3	BenefitsRank[3] - Third most important
99	Did not rank values

0	Not ranked
1	BenefitsRank [1] - Most important

2	BenefitsRank[2] - Second most important
3	BenefitsRank[3] - Third most important
99	Did not rank values

BENE_R_ID - Benefit Ranking Identity

Type	Code
Description	Based on variables in columns BenefitsRank[1], BenefitsRank[2], and BenefitsRank[3]

Benefit Ranking - Aesthetic

0	Not ranked
1	BenefitsRank [1] - Most important
2	BenefitsRank[2] - Second most important
3	BenefitsRank[3] - Third most important
99	Did not rank values

0	Not ranked
1	BenefitsRank [1] - Most important
2	BenefitsRank[2] - Second most important
3	BenefitsRank[3] - Third most important
99	Did not rank values

BENE_R_LIFELESSON - Benefit Ranking Life Lesson

Type	Code
Description	Based on variables in columns BenefitsRank[1], BenefitsRank[2], and BenefitsRank[3]

Benefit Ranking - Aesthetic

0	Not ranked
1	BenefitsRank [1] - Most important
2	BenefitsRank[2] - Second most important
3	BenefitsRank[3] - Third most important

99	Did not rank values

0	Not ranked
1	BenefitsRank [1] - Most important
2	BenefitsRank[2] - Second most important
3	BenefitsRank[3] - Third most important
99	Did not rank values

BENE_R_MENTALWB - Benefit Ranking Mental Well-being

Type	Code
Description	Based on variables in columns BenefitsRank[1], BenefitsRank[2], and BenefitsRank[3]

Benefit Ranking - Aesthetic

0	Not ranked
1	BenefitsRank [1] - Most important
2	BenefitsRank[2] - Second most important
3	BenefitsRank[3] - Third most important
99	Did not rank values

0	Not ranked
1	BenefitsRank [1] - Most important
2	BenefitsRank[2] - Second most important
3	BenefitsRank[3] - Third most important
99	Did not rank values

BENE_R_EXER - Benefit Ranking Exercise

Type	Code
Description	Based on variables in columns BenefitsRank[1], BenefitsRank[2], and BenefitsRank[3]

Benefit Ranking - Aesthetic

0	Not ranked

1	BenefitsRank [1] - Most important
2	BenefitsRank[2] - Second most important
3	BenefitsRank[3] - Third most important
99	Did not rank values

0	Not ranked
1	BenefitsRank [1] - Most important
2	BenefitsRank[2] - Second most important
3	BenefitsRank[3] - Third most important
99	Did not rank values

BENE_R_FUN - Benefit Ranking Fun

Type	Code
Description	Based on variables in columns BenefitsRank[1], BenefitsRank[2], and BenefitsRank[3]

Benefit Ranking - Aesthetic

0	Not ranked
1	BenefitsRank [1] - Most important
2	BenefitsRank[2] - Second most important
3	BenefitsRank[3] - Third most important
99	Did not rank values

0	Not ranked
1	BenefitsRank [1] - Most important
2	BenefitsRank[2] - Second most important
3	BenefitsRank[3] - Third most important
99	Did not rank values

BENE_R_FAMILIAR - Benefit Ranking Familiar

Type	Code
Description	Based on variables in columns BenefitsRank[1], BenefitsRank[2], and BenefitsRank[3]

Benefit Ranking - Aesthetic

0	Not ranked
1	BenefitsRank [1] - Most important
2	BenefitsRank[2] - Second most important
3	BenefitsRank[3] - Third most important
99	Did not rank values

0	Not ranked
1	BenefitsRank [1] - Most important
2	BenefitsRank[2] - Second most important
3	BenefitsRank[3] - Third most important
99	Did not rank values

BENE_R_SOCIAL - Benefit Ranking Social

Type	Code
Description	Based on variables in columns BenefitsRank[1], BenefitsRank[2], and BenefitsRank[3]

Benefit Ranking - Aesthetic

0	Not ranked
1	BenefitsRank [1] - Most important
2	BenefitsRank[2] - Second most important
3	BenefitsRank[3] - Third most important
99	Did not rank values

0	Not ranked
1	BenefitsRank [1] - Most important
2	BenefitsRank[2] - Second most important
3	BenefitsRank[3] - Third most important
99	Did not rank values

BENE_R_SPIRITUAL - Benefit Ranking Spiritual

Type	Code
Description	Based on variables in columns BenefitsRank[1], BenefitsRank[2], and BenefitsRank[3]
Analysis Unit	Individual
Response Unit	Informant

Benefit Ranking - Aesthetic

0	Not ranked
1	BenefitsRank [1] - Most important
2	BenefitsRank[2] - Second most important
3	BenefitsRank[3] - Third most important
99	Did not rank values

0	Not ranked
1	BenefitsRank [1] - Most important
2	BenefitsRank[2] - Second most important
3	BenefitsRank[3] - Third most important
99	Did not rank values

BENE_R_CARE - Benefit Ranking Care

Type	Code
Description	Based on variables in columns BenefitsRank[1], BenefitsRank[2], and BenefitsRank[3]
Analysis Unit	Individual
Response Unit	Informant

Benefit Ranking - Aesthetic

0	Not ranked
1	BenefitsRank [1] - Most important
2	BenefitsRank[2] - Second most important
3	BenefitsRank[3] - Third most important

99	Did not rank values

0	Not ranked
1	BenefitsRank [1] - Most important
2	BenefitsRank[2] - Second most important
3	BenefitsRank[3] - Third most important
99	Did not rank values

BENE_RB_AESTH - Benefit Ranked Aesthetic

Type	Code
Description	Based on variables in columns BenefitsRank[1], BenefitsRank[2], and BenefitsRank[3]
Analysis Unit	Individual
Response Unit	Informant

Benefit Ranked Aesthetic

0	Benefit not ranked
1	Benefit ranked
99	No benefits ranked

0	Benefit not ranked
1	Benefit ranked
99	No benefits ranked

BENE_RB_CREATIVE - Benefit Ranked Creativity

Type	Code
Description	Based on variables in columns BenefitsRank[1], BenefitsRank[2], and BenefitsRank[3]
Analysis Unit	Individual
Response Unit	Informant

Benefit Ranked Aesthetic

0	Benefit not ranked
1	Benefit ranked

99	No benefits ranked

0	Benefit not ranked
1	Benefit ranked
99	No benefits ranked

BENE_RB_TRADITION - Benefit Ranked Tradition

Type	Code
Description	Based on variables in columns BenefitsRank[1], BenefitsRank[2], and BenefitsRank[3]
Analysis Unit	Individual
Response Unit	Informant

Benefit Ranked Aesthetic

0	Benefit not ranked
1	Benefit ranked
99	No benefits ranked

0	Benefit not ranked
1	Benefit ranked
99	No benefits ranked

BENE_RB_FOOD - Benefit Ranked Food

Type	Code
Description	Based on variables in columns BenefitsRank[1], BenefitsRank[2], and BenefitsRank[3]
Analysis Unit	Individual
Response Unit	Informant

Benefit Ranked Aesthetic

0	Benefit not ranked
1	Benefit ranked
99	No benefits ranked

0	Benefit not ranked
1	Benefit ranked
99	No benefits ranked

BENE_RB_ID - Benefit Ranked Identity

Type	Code
Description	Based on variables in columns BenefitsRank[1], BenefitsRank[2], and BenefitsRank[3]

Benefit Ranked Aesthetic

0	Benefit not ranked
1	Benefit ranked
99	No benefits ranked

0	Benefit not ranked
1	Benefit ranked
99	No benefits ranked

BENE_RB_LIFELESSON - Benefit Ranked Life Lesson

Type	Code
Description	Based on variables in columns BenefitsRank[1], BenefitsRank[2], and BenefitsRank[3]
Analysis Unit	Individual
Response Unit	Informant

Benefit Ranked Aesthetic

0	Benefit not ranked
1	Benefit ranked
99	No benefits ranked

0	Benefit not ranked
1	Benefit ranked
99	No benefits ranked

BENE_RB_MENTALWB - Benefit Ranked Mental Well-being

Type	Code
Description	Based on variables in columns BenefitsRank[1], BenefitsRank[2], and BenefitsRank[3]
Analysis Unit	Individual
Response Unit	Informant

Benefit Ranked Aesthetic

0	Benefit not ranked
1	Benefit ranked
99	No benefits ranked

0	Benefit not ranked
1	Benefit ranked
99	No benefits ranked

BENE_RB_EXER - Benefit Ranked Exercise

Type	Code
Description	Based on variables in columns BenefitsRank[1], BenefitsRank[2], and BenefitsRank[3]
Analysis Unit	Individual
Response Unit	Informant

Benefit Ranked Aesthetic

0	Benefit not ranked
1	Benefit ranked
99	No benefits ranked

0	Benefit not ranked
1	Benefit ranked
99	No benefits ranked

BENE_RB_FUN - Benefit Ranked Run

Type	Code
Description	Based on variables in columns BenefitsRank[1], BenefitsRank[2], and BenefitsRank[3]
Analysis Unit	Individual
Response Unit	Informant

Benefit Ranked Aesthetic

0	Benefit not ranked
1	Benefit ranked
99	No benefits ranked

0	Benefit not ranked
1	Benefit ranked
99	No benefits ranked

BENE_RB_FAMILIAR - Benefit Ranked Familiar

Type	Code
Description	Based on variables in columns BenefitsRank[1], BenefitsRank[2], and BenefitsRank[3]
Analysis Unit	Individual
Response Unit	Informant

Benefit Ranked Aesthetic

0	Benefit not ranked
1	Benefit ranked
99	No benefits ranked

0	Benefit not ranked
1	Benefit ranked
99	No benefits ranked

BENE_RB_SOCIAL - Benefit Ranked Social

Type	Code
Description	Based on variables in columns BenefitsRank[1], BenefitsRank[2], and BenefitsRank[3]

Analysis Unit	Individual
Response Unit	Informant

Benefit Ranked Aesthetic

0	Benefit not ranked
1	Benefit ranked
99	No benefits ranked

0	Benefit not ranked
1	Benefit ranked
99	No benefits ranked

BENE_RB_SPIRITUAL - Benefit Ranked Spiritual

Type	Code
Description	Based on variables in columns BenefitsRank[1], BenefitsRank[2], and BenefitsRank[3]
Analysis Unit	Individual
Response Unit	Informant

Benefit Ranked Aesthetic

0	Benefit not ranked
1	Benefit ranked
99	No benefits ranked

0	Benefit not ranked
1	Benefit ranked
99	No benefits ranked

BENE_RB_CARE - Benefit Ranked Care

Type	Code
Description	Based on variables in columns BenefitsRank[1], BenefitsRank[2], and BenefitsRank[3]
Analysis Unit	Individual
Response Unit	Informant

Benefit Ranked Aesthetic

0	Benefit not ranked
1	Benefit ranked
99	No benefits ranked

0	Benefit not ranked
1	Benefit ranked
99	No benefits ranked

BenefitsRank[1]

Type	Code
Description	The statement the respondent ranked as most strongly feeling
Analysis Unit	Individual
Response Unit	Informant

99	Did not rank any statements
A1	I appreciate nature’s sights, smells, sounds, etc.
A10	I appreciate the feeling of familiarity I have with places in nature that I know well
A11	Experiences in nature are an important part of my social life
A12	I feel a connection to something bigger than myself through experiences in nature
A13	I find meaning by caring for nature
A2	Nature inspires me to be creative and express myself
A3	Through experiences of nature, I connect with traditions that are important to me
A4	I rely on nature—a garden, foraging spot, hunting place, etc.—for food
A5	I consider experiences of nature as important to my sense of who I am
A6	I learn important lessons about life from my contact with nature
A7	Experiences of nature contribute to my mental well-being

A8	Time in nature helps me stay in shape and get my exercise
A9	I spend time in nature for leisure and fun

99	Did not rank any statements
A1	I appreciate nature's sights, smells, sounds, etc.
A10	I appreciate the feeling of familiarity I have with places in nature that I know well
A11	Experiences in nature are an important part of my social life
A12	I feel a connection to something bigger than myself through experiences in nature
A13	I find meaning by caring for nature
A2	Nature inspires me to be creative and express myself
A3	Through experiences of nature, I connect with traditions that are important to me
A4	I rely on nature—a garden, foraging spot, hunting place, etc.—for food
A5	I consider experiences of nature as important to my sense of who I am
A6	I learn important lessons about life from my contact with nature
A7	Experiences of nature contribute to my mental well-being
A8	Time in nature helps me stay in shape and get my exercise
A9	I spend time in nature for leisure and fun

BenefitsRank[2]

Type	Code
Description	The statement the respondent ranked as second most strongly feeling
Analysis Unit	Individual
Response Unit	Informant

99	Did not rank any statements

A1	I appreciate nature’s sights, smells, sounds, etc.
A10	I appreciate the feeling of familiarity I have with places in nature that I know well
A11	Experiences in nature are an important part of my social life
A12	I feel a connection to something bigger than myself through experiences in nature
A13	I find meaning by caring for nature
A2	Nature inspires me to be creative and express myself
A3	Through experiences of nature, I connect with traditions that are important to me
A4	I rely on nature—a garden, foraging spot, hunting place, etc.—for food
A5	I consider experiences of nature as important to my sense of who I am
A6	I learn important lessons about life from my contact with nature
A7	Experiences of nature contribute to my mental well-being
A8	Time in nature helps me stay in shape and get my exercise
A9	I spend time in nature for leisure and fun

99	Did not rank any statements
A1	I appreciate nature’s sights, smells, sounds, etc.
A10	I appreciate the feeling of familiarity I have with places in nature that I know well
A11	Experiences in nature are an important part of my social life
A12	I feel a connection to something bigger than myself through experiences in nature
A13	I find meaning by caring for nature
A2	Nature inspires me to be creative and express myself

A3	Through experiences of nature, I connect with traditions that are important to me
A4	I rely on nature—a garden, foraging spot, hunting place, etc.—for food
A5	I consider experiences of nature as important to my sense of who I am
A6	I learn important lessons about life from my contact with nature
A7	Experiences of nature contribute to my mental well-being
A8	Time in nature helps me stay in shape and get my exercise
A9	I spend time in nature for leisure and fun

BenefitsRank[3]

Type	Code
Description	The statement the respondent ranked as third most strongly feeling
Analysis Unit	Individual
Response Unit	Informant

99	Did not rank any statements
A1	I appreciate nature’s sights, smells, sounds, etc.
A10	I appreciate the feeling of familiarity I have with places in nature that I know well
A11	Experiences in nature are an important part of my social life
A12	I feel a connection to something bigger than myself through experiences in nature
A13	I find meaning by caring for nature
A2	Nature inspires me to be creative and express myself
A3	Through experiences of nature, I connect with traditions that are important to me
A4	I rely on nature—a garden, foraging spot, hunting place, etc.—for food

A5	I consider experiences of nature as important to my sense of who I am
A6	I learn important lessons about life from my contact with nature
A7	Experiences of nature contribute to my mental well-being
A8	Time in nature helps me stay in shape and get my exercise
A9	I spend time in nature for leisure and fun

99	Did not rank any statements
A1	I appreciate nature’s sights, smells, sounds, etc.
A10	I appreciate the feeling of familiarity I have with places in nature that I know well
A11	Experiences in nature are an important part of my social life
A12	I feel a connection to something bigger than myself through experiences in nature
A13	I find meaning by caring for nature
A2	Nature inspires me to be creative and express myself
A3	Through experiences of nature, I connect with traditions that are important to me
A4	I rely on nature—a garden, foraging spot, hunting place, etc.—for food
A5	I consider experiences of nature as important to my sense of who I am
A6	I learn important lessons about life from my contact with nature
A7	Experiences of nature contribute to my mental well-being
A8	Time in nature helps me stay in shape and get my exercise
A9	I spend time in nature for leisure and fun

Unranked

Type	Numeric (Integer)
Description	If no statements ranked = 1; If one or more statements ranked = 0
Analysis Unit	Individual
Response Unit	Informant

UA v FA

Type	Numeric (Integer)
Description	Value factor dependent variable assignments for participants. 1 = Nurture and Recreation factor; 2 = unassigned factor; 3 = Inspiration and Nourishment Factor.
Analysis Unit	Individual
Response Unit	Informant

Zip - Zip Code

Type	Numeric (Integer)
Numeric Details	Decimals: 0
Description	Raw entries for respondents’ zip codes, used to determine urban/rural status. 99 = missing value
Analysis Unit	Individual
Response Unit	Informant

LSAD10

Type	Code
Description	2010 U.S. census statistical area codes based on zip codes using ArcGIS geocoding tool; missing = blank

Analysis Unit	Individual
Response Unit	Informant

75	Urban Area
76	Urbanized Cluster
99	Rural Area

75	Urban Area
76	Urbanized Cluster
99	Rural Area

UATYP10

Type	Code
Description	2010 U.S. census statistical area codes based on zip codes using ArcGIS geocoding tool; missing = blank
Analysis Unit	Individual
Response Unit	Informant

99	Missing value
C	Urbanized cluster
R	Rural area
U	Urban area

99	Missing value
C	Urbanized cluster
R	Rural area
U	Urban area

GeographyCat - Geography Cat

Type	Code
Description	Category based on column UATYP10

Geography Cat

99	Missing value
Not urban area	R or C

Urban area	U

99	Missing value
Not urban area	R or C
Urban area	U

GeographyCatNum

Type	Code
Description	Numerical Variable based on column UATYP10

0	R or C
1	U
99	Missing value

0	R or C
1	U
99	Missing value

GeographyCatNumInverse

Type	Code
Description	Numerical Variable based on column UATYP10; Inverse of GeographyCatNum column
Analysis Unit	Individual
Response Unit	Informant

1	R or C
99	Missing value
0	U

1	R or C
99	Missing value
0	U

Age

Type	Numeric (Integer)
Numeric Details	Decimals: 0

Description	Respondents age in years, calculated from BirthYear; 0 = missing
Analysis Unit	Individual
Response Unit	Informant

BirthYear

Type	Date
Description	Raw entries for respondents' birth years. 0 = missing.
Analysis Unit	Individual
Response Unit	Informant

GenderCat

Type	Code
Description	Categorical labels for raw entries from Gender column
Analysis Unit	Individual
Response Unit	Informant

99	Missing or non-binary
Female	Female
Male	Male

99	Missing or non-binary
Female	Female
Male	Male

GenderCatNum

Type	Code
Description	Numeric variables corresponding to GenderCat.
Analysis Unit	Individual
Response Unit	Informant

0	Male
1	Female
99	Missing value or Non-binary

0	Male
1	Female

99	Missing value or Non-binary

GenderCatNumInverse

Type	Code
Description	Inverse coding of GenderCatNum
Analysis Unit	Individual
Response Unit	Informant

0	Female
1	Male
99	Missing or Non-binary

0	Female
1	Male
99	Missing or Non-binary

Gender

Type	Code
Description	Raw entries for respondents' gender
Analysis Unit	Individual
Response Unit	Informant

99	Missing value
A1	Man
A2	Woman
A3	Non-binary

99	Missing value
A1	Man
A2	Woman
A3	Non-binary

Race/EthnicityCatNum

Type	Code
Description	Numeric variables corresponding to Race/EthnicityCat
Analysis Unit	Individual

Response Unit	Informant

0	White
1	All but White
99	Missing value

0	White
1	All but White
99	Missing value

Race/EthnicityCatNumInverse

Type	Code
Description	Inverse coding of Race/EthnicityCatNum
Analysis Unit	Individual
Response Unit	Informant

0	All but White
1	White
99	Missing value

0	All but White
1	White
99	Missing value

Race/EthnicityCat

Type	Code
Description	Categorical labels condensing the raw entries in Race/EthnicitX[SQ00X] fields into two bins
Analysis Unit	Individual
Response Unit	Informant

99	Missing value
All but white	Respondents who checked any other combination of Race/EthnicitX[SQ00X] fields
White	Respondents who checked only RaceEthnicit7[SQ007]

99	Missing value

All but white	Respondents who checked any other combination of Race/EthnicitX[SQ00X] fields
White	Respondents who checked only RaceEthnicit7[SQ007]

RaceEthnicit1[SQ001] - American Indian or Alaskan Native

Type	Text
Description	Which categories do you most closely identify with? Select all that apply. Y = yes, identifies as that race/ethnicity; Blank = no, does not identify as that race/ethnicity or blank
Analysis Unit	Individual
Response Unit	Informant

RaceEthnicit1[SQ002] - Asian

Type	Text
Description	Which categories do you most closely identify with? Select all that apply. Y = yes, identifies as that race/ethnicity; Blank = no, does not identify as that race/ethnicity or blank
Analysis Unit	Individual
Response Unit	Informant

RaceEthnicit1[SQ003] - Black or African American

Type	Text
Description	Which categories do you most closely identify with? Select all that apply. Y = yes, identifies as that race/ethnicity; Blank = no, does not identify as that race/ethnicity or blank
Analysis Unit	Individual
Response Unit	Informant

RaceEthnicit1[SQ004] - Hispanic, Latino, or Spanish

Type	Text
Description	Which categories do you most closely identify with? Select all that apply. Y = yes, identifies as that race/ethnicity; Blank = no, does not identify as that race/ethnicity or blank
Analysis Unit	Individual
Response Unit	Informant

RaceEthnicit1[SQ005] - Middle Eastern or North African

Type	Text
Description	Which categories do you most closely identify with? Select all that apply. Y = yes, identifies as that race/ethnicity; Blank = no, does not identify as that race/ethnicity or blank
Analysis Unit	Individual
Response Unit	Informant

RaceEthnicit1[SQ006] - Native Hawaiian or Pacific Islander

Type	Text
Description	Which categories do you most closely identify with? Select all that apply. Y = yes, identifies as that race/ethnicity; Blank = no, does not identify as that race/ethnicity or blank
Analysis Unit	Individual
Response Unit	Informant

RaceEthnicit7[SQ007] - White

Type	Text
Description	Which categories do you most closely identify with? Select all that apply. Y = yes, identifies as that race/ethnicity; Blank = no, does not identify as that race/ethnicity or blank
Analysis Unit	Individual
Response Unit	Informant

IncomeCat

Type	Code
Description	Categorical labels condensing raw entries from Income into bins; missing = 99
Analysis Unit	Household
Response Unit	Informant

99	Missing value
Above Median Range	A5, A6, A7 A8
Below Median Range	A1, A2, or A3
Median Range	A4

99	Missing value
Above Median Range	A5, A6, A7 A8
Below Median Range	A1, A2, or A3
Median Range	A4

IncomeCatNum

Type	Code
Description	Numeric variable corresponding to IncomeCat
Analysis Unit	Household
Response Unit	Informant

1	Below median income
2	Median range income
3	Above median income
99	Missing value

1	Below median income
2	Median range income
3	Above median income
99	Missing value

IncomeCatNumInverse

Type	Code
Description	Inverse of IncomeCatNum
Analysis Unit	Household
Response Unit	Informant

1	Above median income
2	Median income range
3	Below median income
99	Missing value

1	Above median income
2	Median income range

3	Below median income
99	Missing value

Income

Type	Code
Description	Raw entries for "In 2019, about what was your total household income, before taxes? Please count income from all members of your household, and from all sources."
Analysis Unit	Household
Response Unit	Informant

99	Missing value
A1	Less than $10,000
A2	$10,000-$24,999
A3	$25,000-$49,000
A4	$50,000-$74,999
A5	$75,000-$99,999
A6	$100,000-$149,999
A7	$150,000-$199,999
A8	Greater than $200,000

99	Missing value
A1	Less than $10,000
A2	$10,000-$24,999
A3	$25,000-$49,000
A4	$50,000-$74,999
A5	$75,000-$99,999
A6	$100,000-$149,999
A7	$150,000-$199,999
A8	Greater than $200,000

EmploymentCatNum - Employment CatNum

Type	Code
Description	Numeric variable corresponding to EmploymentCat

Analysis Unit	Individual
Response Unit	Informant

Employment CatNum

0	All other fields
1	Lost job
99	Missing value or marked two incompatible values (e.g. lost job and not applicable)

0	All other fields
1	Lost job
99	Missing value or marked two incompatible values (e.g. lost job and not applicable)

EmploymentCatNumInverse

Type	Code
Description	Inverse of EmploymentCatNum
Analysis Unit	Individual
Response Unit	Informant

0	Lost job
1	All other fields
99	Missing value or marked two incompatible values (e.g. lost job and not applicable)

0	Lost job
1	All other fields
99	Missing value or marked two incompatible values (e.g. lost job and not applicable)

EmploymentCat - Employment Categories

Type	Code
Description	Categorical labels condensing the raw entries in Employment[SQ00X] fields into two bins
Analysis Unit	Individual
Response Unit	Informant

Employment Categories

99	Missing value or marked two incompatible values (e.g. lost job and not applicable)
All other fields	Respondents who retained their job in any form
Lost job	Respondents that lost a job - "Y" Employment[SQ001]

99	Missing value or marked two incompatible values (e.g. lost job and not applicable)
All other fields	Respondents who retained their job in any form
Lost job	Respondents that lost a job - "Y" Employment[SQ001]

Employment[SQ001] - Lost Job

Type	Text
Description	Responsed "I lost my job " to question "Since the coronavirus outbreak started (March 11th), have you experienced a loss of income and/or job? Please select all that apply."
Analysis Unit	Individual
Response Unit	Informant

Employment[SQ002] - Hour Reduction

Type	Text
Description	Responsed "I have had a reduction in hours at my job, reducing my income " to question "Since the coronavirus outbreak started (March 11th), have you experienced a loss of income and/or job? Please select all that apply."
Analysis Unit	Individual
Response Unit	Informant

Employment[SQ003] - Furloughed

Type	Text
Description	Responsed "I have been furloughed, reducing my income " to question "Since the coronavirus outbreak started (March 11th), have you experienced a loss of income and/or job? Please select all that apply."
Analysis Unit	Individual
Response Unit	Informant

Employment[SQ004] - Work From Home

Type	Text
Description	Responsed "Continuing to work from home, same hours and income" to question "Since the coronavirus outbreak started (March 11th), have you experienced a loss of income and/or job? Please select all that apply."
Analysis Unit	Individual
Response Unit	Informant

Employment[SQ005] - Work in Person

Type	Text
Description	Responsed "Continuing to work in person, same hours and income" to question "Since the coronavirus outbreak started (March 11th), have you experienced a loss of income and/or job? Please select all that apply."
Analysis Unit	Individual
Response Unit	Informant

Employment[SQ006] - Not Applicable

Type	Text
Description	Responsed "Not applicable " to question "Since the coronavirus outbreak started (March 11th), have you experienced a loss of income and/or job? Please select all that apply."
Analysis Unit	Individual
Response Unit	Informant
