# Supplementary material for: COVID-19 and human-nature relationships: Vermonters’ activities in nature and associated nonmaterial values during the pandemic
Source: PLoS One. 2020 Dec 11;15(12):e0243697. doi: 10.1371/journal.pone.0243697 (PMC7732125; doi:10.1371/journal.pone.0243697)
Supplement: S1 File — (PDF) [file pone.0243697.s010.pdf]

As we in Vermont continue with social distancing, we're curious: does experiencing nature figure into your life right now? If so, how?

Hopefully, this survey can help policymakers and researchers understand how to make policy decisions that account for the role nature may have during events like COVID-19, now and in the future.

We'll enter you into a drawing for one of twenty $50 prepaid Mastercards if you fill out two 10-minute surveys: one now, and one after the COVID-19 restrictions are lifted.

Detailed Study Information

Thank you for your interest in our survey, which will allow you to share your experiences with nature during and after COVID-19-related restrictions. This research study is being conducted by researchers at the University of Vermont. You must be 18 or older to participate.

Purpose This study explores whether COVID-19 restrictions impact how Vermont residents experience nature. We hope that this information can help researchers and decision-makers understand the role that nature-related experiences play in crisis situations.

Study Procedures This study involves two short surveys. The first survey includes questions about you and your experiences of nature, then requests your email address. We will send the second survey to that address. The second survey includes a few additional questions about your experience of nature. Both surveys will take 10 minutes.

Compensation If you participate in both rounds of the survey, you will be entered in a drawing for one of twenty $50 prepaid Mastercards.

Benefits There will be no other direct benefits to you for taking part in this survey. We hope that the information from this study may benefit others now and in the future.

Risks We do not anticipate that your participation will entail any risks. We will do our best to protect the information we collect from you during this study. We will not collect any information that will identify you to further protect your confidentiality and avoid any potential risk for an accidental breach of confidentiality.

Costs There will be no costs to you for participation in this research study.

Confidentiality All information collected about you will be stored with a code name or number so that we are able to match you to your answers. We need to be able to match you to your answers in order to send you the appropriate follow-up email. We will use your email address only to conduct the gift card drawing and to link your responses to the two surveys. We will not use your email address for any other purpose and will not distribute it to anyone beyond our small research team.

Voluntary Participation/Withdrawal Taking part in this study is voluntary. You are free to not answer any questions or withdraw at any time. If you decide to take part in the study, you can change your mind later and withdraw. If you withdraw before completing the survey, we will delete your responses from our records.

B1. Please tell us how you first found this survey.

Front Porch Forum
A mailing from an organization
A friend or colleague sent it to me
Other

Other

B2. What organization?

C1. Compared to this time last year, how much time are you spending experiencing nature in each of these places right now? On the slider, 1 means much less time, 7 means much more time, and 4 means about the same amount of time.

My home or yard | Much less time | Much more time

A short walk beyond my home | Much less time | Much more time

Farther than a walk but within a 30-minute drive of home | Much less time | Much more time

Farther than a 30-minute drive from home | Much less time | Much more time

Remotely, through things like books, photographs, television programs, and/or the internet | Much less time | Much more time

C2.

C3. Compared to this time last year, how frequently are you participating in these activities in a typical month?

I don't do this activity	Much less	Less	Somew hat less	Same amount	Somewh at more	More	Much more

C4. Are there any other ways that the COVID-19 restrictions have altered your participation in these activities? If so, tell us how.

D1. We'd like to know more about how you relate to nature right now, <u>during the COVID-19 restrictions</u>. Please read each of the following statements and indicate your level of agreement: 1 means strongly disagree, 7 means strongly agree, and 4 means neither agree nor disagree.

I appreciate nature's sights, smells, sounds, etc. | Strongly disagree | Strongly agree

Nature inspires me to be creative and express myself | Strongly disagree | Strongly agree

Through experiences of nature, I connect with traditions that are important to me | Strongly disagree | Strongly agree

I rely on nature—a garden, foraging spot, hunting place, etc.—for food | Strongly disagree | Strongly agree

I consider experiences of nature as important to my sense of who I am | Strongly disagree | Strongly agree

I learn important lessons about life from my contact with nature | Strongly disagree | Strongly agree

Experiences of nature contribute to my mental well-being | Strongly disagree | Strongly agree

Time in nature helps me stay in shape and get my exercise | Strongly disagree | Strongly agree

I spend time in nature for leisure and fun | Strongly disagree | Strongly agree

I appreciate the feeling of familiarity I have with places in nature that I know well | Strongly disagree | Strongly agree

Experiences in nature are an important part of my social life | Strongly disagree | Strongly agree

I feel a connection to something bigger than myself through experiences in nature | Strongly disagree | Strongly agree

I find meaning by caring for nature | Strongly disagree | Strongly agree

D2. **Are any of those statements truer for you now, <u>during the COVID-19 restrictions</u>, than they were before the restrictions? If yes, drag-and-drop up to three statements that feel truer now into the right-hand column.**

Place the statement you most strongly feel this for first, then the one you feel it next most strongly for second, and the one you feel it third most strongly for third.

I appreciate nature's sights, smells, sounds, etc.	☐☐☐
Nature inspires me to be creative and express myself	☐☐☐
Through experiences of nature, I connect with traditions that are important to me	☐☐☐
I rely on nature—a garden, foraging spot, hunting place, etc.—for food	☐☐☐
I consider experiences of nature as important to my sense of who I am	☐☐☐
I learn important lessons about life from my contact with nature	☐☐☐
Experiences of nature contribute to my mental well-being	☐☐☐
Time in nature helps me stay in shape and get my exercise	☐☐☐
I spend time in nature for leisure and fun	☐☐☐
I appreciate the feeling of familiarity I have with places in nature that I know well	☐☐☐
Experiences in nature are an important part of my social life	☐☐☐
I feel a connection to something bigger than myself through experiences in nature	☐☐☐
I find meaning by caring for nature	☐☐☐

D3. Feel free to explain your selections if you like. Why did you choose the one(s) you chose as more important to you now, <u>during COVID-19 restrictions</u>?

E1. Please answer the following questions about your experience of nature given the current COVID-19 restrictions.

Are the ways you experience nature important to how you are coping during the COVID-19 restrictions? I Not at all important to how I am coping I Extremely important to how I am coping	
Are the COVID-19 restrictions influencing how much experience of nature you have compared to this same time last year?I Because of the restrictions I've had significantly less experiences of nature I Because of the restrictions I've had significantly more experiences of nature	
Do you think that restrictions related to COVID-19 positively contribute to slowing the spread of the virus? I I don't think that restrictions slow the spread of COVID at all I I think that restrictions certainly slow the spread of COVID	
Would you be willing to accept increased restrictions that influence your experience of nature and nature activities to help slow the spread of COVID-19? I I would certainly not accept any further restrictions on nature activities I I would certainly accept further restrictions on nature activities	

E2. Is there anything else you would like to tell us about how the COVID-19 restrictions relate to your experience of nature?

F1. **Please select the picture below that best describes your relationship with the natural environment. Answer spontaneously with what first comes to your mind.**

F2. **We have one final question about nature and COVID-19. Is your experience of nature <u>more</u> or <u>less</u> meaningful to you right now, during the COVID-19 restrictions? If so, can you tell us why or why not?**

G1. **In what zip code do you live?**

G2. **In what year were you born?**

G3. **What is your gender?**

Man
Woman
Non-binary

G4. **Which categories do you most closely identify with? Select all that apply.**

American Indian or Alaskan Native
Asian
Black or African American
Hispanic, Latino, or Spanish
Middle Eastern or North African
Native Hawaiian or Pacific Islander
White
Other

Other

G5. **Where would you place yourself along the political spectrum?**

Very conservative
Conservative
Slightly conservative
Moderate
Slightly liberal
Liberal
Very liberal

G6. **In 2019, about what was your total household income, before taxes? Please count income from all members of your household, and from all sources.**

Less than $10,000
$10,000-$24,999
$25,000-$49,000
$50,000-$74,999
$75,000-$99,999
$100,000-$149,999
$150,000-$199,999
Greater than $200,000

G7. **Since the coronavirus outbreak started (March 11th), have you experienced a loss of income and/or job? Please select all that apply.**

I lost my job
I have had a reduction in hours at my job, reducing my income
I have been furloughed, reducing my income
Continuing to work from home, same hours and income
Continuing to work in person, same hours and income
Not applicable

G8. **We would like to follow up with you about your experiences in nature once the COVID-19 restrictions are lifted. Please provide <u>your email address</u> below to receive the follow-up survey. We will not use your email address for any other purpose and will not distribute it to anyone beyond our small research team. After we complete the second survey, you will be entered into a drawing for one of 20 $50 prepaid Mastercards.**

G9. **Please re-enter your email, just to be sure!**

Thank you for taking our survey! We will send a follow-up survey to the email address you provided after COVID-19 restrictions are lifted. Your answers will help us to understand people's experience of nature during these times.
