# Supplementary material for: COVID-19 and human-nature relationships: Vermonters’ activities in nature and associated nonmaterial values during the pandemic
Source: PLoS One. 2020 Dec 11;15(12):e0243697. doi: 10.1371/journal.pone.0243697 (PMC7732125; doi:10.1371/journal.pone.0243697)
Supplement: Attachment — Submitted filename: Responses to Reviewers.docx [file pone.0243697.s012.docx]

Dear Dr. Martin-Ortega,

My co-authors and I are grateful for your, and our two reviewers’, thoughtful comments. We have undertaken a substantial revision of our manuscript to address the important questions and suggestions that the review process raised. Our full responses to each editor, reviewer, and journal comment are compiled in the **Reviewer Response Table** at the end of this letter. We also summarize the major changes we have made in the following paragraphs. These fall into two main areas: 1) revised statistical analysis; and 2) expanded discussion of theory and policy implications.

Following on Reviewer 2’s urging to consider measures of model goodness of fit beyond the Pseudo R-square statistic, we present an updated and more conservative statistical analysis. We now limit our interpretation of six of our seven models to the largest levels of the dependent variables only (per Petrucci 2009), and engage with the rare events in logistic regression literature (Firth 1993, King and Langche 2001, Kosmidis and Firth 2011, and others). We discuss the rationale for this decision and provide supporting citations in both Methods and Discussion (Limitations) sections. We also provide a comment on the equity and policy implications of taking this conservative approach.

Following on your own Editor comments, we have developed our treatment of the theory implications of this work. In the Introduction, we better integrate our research into the literature on human-nature relationships and disasters, drawing on Rung et al. 2011, Derks et al. 2020, and others. We also situate our approach within the emerging literature on relational values (e.g. Klain et al. 2017) and comment on the potential to integrate this literature with value-change research. We also elaborate on our work in the context of Fish et al. 2016’s framework relating cultural practices in nature to values from nature. In the Discussion, we more thoroughly discuss the policy implications of this work in an equity context. We investigate the rare events logistic regression literature noted above in terms of our study’s relevance and shortcomings for informing equitable natural resource management policy. We also draw briefly on the policy sciences literature to support our observation of diverse values from shared experiences of natural resources (Clark and Vernon 2016, Morse and Clark 2019).

Additionally, we have made a number of smaller changes for clarity and to ensure that our formatting is consistent with PLOS ONE’s requirements. We are eager for your, and other editorial team members’, impressions of this revised manuscript, and much appreciative of your time and consideration.

Sincerely,

Joshua Morse

Tatiana Gladkikh

Diana Hackenburg

Rachelle Gould

**REVIEWER RESPONSE TABLE**

| **Reviewer Comment** | **Response** |
| --- | --- |
| **EDITOR COMMENTS** | |
| “Firstly, you need to explain what do you mean by "dynamism" of human-nature relationships and how specifically that is reflected in your study design. Do you mean just change? Or is it a more complex concept than that?” | We clarified wording, replacing “dynamism” with “the potential for change” and similar phrases appropriate to specific context, throughout. |
| “Secondly, the discussion mostly describes patterns of behaviour detected in the survey, but I miss a deeper/more profund discussion on what does this fundamentally means in terms of our understanding of human nature relationships.” | We appreciate this thoughtful, big-picture comment. We have taken two steps to highlight the deeper implications of our work for understanding human-nature relationships.  First, we significantly expand our treatment of how this paper adds to pandemic and disaster research on human-nature relationships (Introduction). Here, we further highlight the need to understand linkages between previously documented pandemic-driven changes in outdoor activity, and the underlying values around nature that these changes may impact, and incorporate additional relevant literature (Rice et al. 2020, Rung et al. 2011, Derks et al. 2020, and Venter et al. 2020).  Second, we update our Discussion section to more clearly characterize changes in terms of theory, the role of values, and socio-demographic influences. We make explicit the theoretical implications of our work especially in the first two paragraphs of this section, concluding by flagging our intention that this paper help draw attention to the need for better understanding of the links between diverse cultural practices and environmental values (per Fish et al. 2016). |
| “…most of the discussion describes the behavioural changes (which are, by the way, well discussed/described and referenced) and not so much the complexity and dynamism of such relationships and I believe this could be done more/further developed.” | We have expanded our treatment of the role that behavior change and value change may have for our understanding of human-nature relationships. Drawing on Fish et al. 2016 in the opening paragraph of our COVID and Nature-Based Activities section of the Discussion, we clarify the role of activities in nature as a centerpiece of human-nature relationships and one pathway for evolution in those relationships. Drawing on Daniels et al. 2012, Chan et al. 2016, and Klain et al. 2017, we expand the opening paragraph of our COVID and Values From Nature section of the Discussion to introduce the idea of relational values as a new dimension of human-nature relationships. We suggest that research on value change be integrated into the emerging relational values literature. |
| “I would like to see [the Nurture/Recreation vs Inspirational values aspects of your analysis] further developed with respect to what does it mean for our understanding of the complex relationships.” | We have developed our interpretation of the importance of two distinct factors in the range of values we sampled in terms of policy consequence in the Conclusion and Policy Recommendations section. We draw on prior research on diverse values in a natural resources context with strong management implications (Morse and Clark 2019; Clark and Vernon 2016) to underscore this point. |
| Thirdly, and still in relation to rising to the promise made at the introduction, I find that most of the recommendations for policy making made in the conclusion section are quite generic (and somewhat well-known) and could be made more specific/insightful with respect to disaster management and in the context of the pandemic. | We very much appreciate this invitation to more carefully reflect on our work’s policy implications. We have expanded the Conclusion section (now Conclusion and Policy Recommendations) considerably, with a more nuanced treatment of the topic. In particular, we investigate how our survey methods and updated statistical analysis (see responses to Reviewer 2 comments, below) both off a useful template for policy relevant research during the pandemic, and highlight the equity shortcomings to common social science approaches that will need to be considered in any application of this—or similar—work to decision making. |
| “The methodology section does not explain sufficiently clearly/explicitly what is the purpose of regressing the dependent variables against the factor/values identified in the factor analysis…You should provide some rationale of this process in the section that you call ‘Analysis of values’.” | We are grateful for this invitation to clarify our choice of analytical methods as pertaining to our research questions. We consider this question relevant to both the values analysis as specified by the Editor, and also the activities analysis. Accordingly, we have added text to the “Analysis of Activities” and “Analysis of Values” sub-sections that discusses: 1) why our specific research questions were best approached with multiple logistic regression; and 2) how these questions and methods contribute to larger discussion around demographics, environmental activities and values, and equity. We further expand on the equity implications of both our findings, and our choice of methods, in the Limitations section. |
| “It would be clearer if you defined the two values (Nurture/Recreation; Inspiration/Nourishment) when you first present the factor analysis (in the Analysis of values section) and not in the results section…more clarity and earlier signposting of the prupose of this part of the analysis should help…” | We appreciate this recommendation for clarity and have made the suggested changes. |
| “…you need to explain more specifically what the results mean/imply…Rather than just refering to the table, it would help if you developed in the text more what are those findings and what they mean.” | We have included more thorough interpretation of our results throughout the discussion section. |
| “In the limitations section, consider referering to self-selection bias. how was the survey presented to participants? could it be that those more active in recreational activities felt more inclined to respond to the survey? How was the framing/context of the survey.” | We have expanded our discussion of potential biases in our sampling approach, per this comment and comments from Reviewer 2. Additionally, we have updated our methods to include the language of the list serve posting we used, and template language from the recruitment email we used to recruit community partner organizations. This should better allow readers to assess the risks of sampling bias. |
| “You make some reference to Vermont being quite particular in the US context. what about the possibility of your study being more or less relevant more broadly.” | We have added a clarification that our results are likely indicative of predominantly rural, White areas of the country, but should be generalized with caution in other contexts. |
| “In the methods section, you explain that the geographical location of participants was partly recorded. This doesn’t seem to have been used in the analysis (was it used to differentiate urban/rural?). Could there been room for a more-fine grained spatial analysis, e.g. with relation to access to sites, etc.” | A finer-grained analysis of participant geographies, while possible, was beyond the scope of this study. Two factors are important in this consideration. First, leaving our analysis of geography at the level of urban/rural delineation is consistent with some of the emerging literature on nature activity and the pandemic (e.g. Rice et al. 2020a) geared towards large-scale environmental management. Thus, we felt that this level of analysis was an adequate contribution to the literature in its own right. Second, a deeper investigation of our geographic data would certainly merit a second paper owing to the complexity of outdoor access in Vermont. The state’s predominantly privately-owned landscape, rural character, and complex ethic of land use are all complicating factors that would require additional data collection to rigorously address. |
| **REVIEWER 1 COMMENTS** | |
| “Line 345 Is 'phenomenon' the right word? Would 'result' or something similar be better to avoid methodological confusion?” | We have revised our wording per the Reviewer’s recommendation. |
| **REVIEWER 2 COMMENTS** | |
| “Overall comment. Quite a bit of recreation research related to activity and COVID-19 has been conducted to date. Some of this is cited, but additional discussion of this research base is merited in the introduction [consider Venter et al 2020; Derks et al 2020; O’Connell et al 2020].” | We are grateful to this reviewer for alerting us to references of which we were unaware. We have added these references to the paper and offered clarification as to the importance of studying both increased outdoor activity in response to the pandemic, as well as the values that may underlie this activity. |
| “Line 40. You may want to look to the work done following Hurricane Katrina for some insight in this area… You cite Rojas et al. (2017), but there is a larger body of work out there that directly speaks to this area you claim to be lacking [consider Rung et al 2011].” | We are grateful to Reviewer 2 for drawing out attention to Rung et al. 2011, which we have incorporated into our introduction. After reviewing the body of literature that cites Rung et al. 2011, we determined that the majority is more narrowly focused on psychological well-being, and leisure, and thus narrower in scope than we believe is relevant to our analysis. |
| “Lines 47-63. Given the scope of this paper, the authors should be more specific in their discussion of ecosystem services, namely, specifying that many of these values/benefits are recreational ecosystem services, a sub-class of CES [consider Rice et al 2020a & 2020b].” | We are thankful for the recommendation of the recreational ecosystems services (RES) literature. Rice et al 2020a & 2020b provide a valuable insight in management of protected areas for recreation. We have expanded our characterization of ecosystem services, the human-nature relationships that grant access to them, and the work we cite defining these nuances, in response.  However, we politely disagree with the suggested recreational ecosystem services (RES) framework and classification of some of the discussed values/benefits as RES as described in Rice et al (2020b). We argue that CES as a whole, not just RES, are co-produced and co-created through diverse interactions of people with ecosystems (Chan et al., 2016; Fish et al., 2016). We cannot conclude whether some of the reported values were associated primarily or solely with outdoor recreation, as labeling of some values as sub-group of recreation would imply. Accordingly, we worry that grouping some of the values in our study as specific to recreational interactions might mask the diversity of values people associate with ecosystems, and the relationships that undergird them (see Biedenweg et al., 2019). In planned follow-up research, we hope to more closely assess the connection between specific activities (recreational and otherwise) and environmental values. |
| “Line 107. How many other listserves and social media platforms were used in the data collection?” | We have updated the methods with a count of community partner organizations who distributed the survey. |
| “Line 113. What organization?” | We prefer not to disclose the list of organizations by name, since our IRB does not specify whether we can do so. |
| “Lines 110-115. I would strongly encourage the authors to move this paragraph to the results section.” | We have moved the paragraph, per Reviewer 2’s recommendation. |
| “Line 120. How were these 15 activities selected? From previous research? Or the OIA or other list?” | We have added details and a supporting citation describing our method for selecting activities. |
| Lines 164-166. A citation is needed here; Lines 188-189. Citation needed. | We have added a citation on the suitability of multinomial logistic regression for analyzing datasets with three un-ordered levels of the dependent variable (RE line 164: Petrucci 2009) and the use of oblique rotation in Factor analysis (RE line 188: Yong and Pearce 2013). |
| “Line 202. A little confused here. Define “any combination”. Does this mean that if a participant designated one of the Factor 1 values as more important, they would be coded as 1 in the factor 1 dependent variable?”  “Line 202. I am similarly confused as to why values that were not included in either factor are included in both analyses: “we classified participants who ranked any combination of factor 1 values and values unassigned to either factor as more important during the COVID-19 restrictions as “1””. Language at the start of the paragraph does not suggest this. Additionally, it is unclear to the reader how many of those coded as “1” in the factor 2 dependent variable did not actually rank factor 2 values in their top three, but instead ranked unassigned values in their top three. Looking at Figure 2, the latter groups seems larger than the former. This has serious implications in your discussion of these results, and causes question towards the validity of this study. I understand that these unassigned values “loaded strongly with both factors,” but their inclusion in BOTH factors is not warranted and seems to undermine the validity of the study.” | We have clarified the language pertaining to assigning respondents to Factor 1 or Factor 2 dependent variable levels, and added a table (Table 3) further illustrating these assignments, in response. We offer additional details in response to Reviewer 2’s comments below.  We treated unassigned values as neutral when categorizing respondents to the factor dependent variable, because these values loaded onto both factors. It appears that Reviewer 2's concern that this would lead to categorizing respondents who ranked only unassigned values to a factored dependent variable level arises from misunderstanding this categorization. With unassigned values functioning as neutral for the dependent variable, only respondents who ranked factored values (either alone or alongside unassigned values) would be categorized in the corresponding factor's dependent variable level. This approach is consistent with our interest in potential demographic differences between respondents who exclusively prioritized value of one factor over the other.  However, pre Reviewer 2’s broad interest in participants who were not grouped into either factor’s dependent variable level, we updated our values regression. Our updated approach uses a single multinomial logistic regression with a three-level dependent variable reflecting whether participants were grouped into factor 1, factor 2, or unassigned to either value. |
| “Line 238. These Pseudo R-squares are markedly low. I would recommend 1) discussing this statistic in the methods section (consider looking at Sapra (2014)), 2) being forthright about the low R2 in the results section, and 3) increasing the discussion of the limitations of this statistic and how these low R2s might impact the gravity of your findings in the limitations section [consider Sapra 2014].” | We are grateful for Reviewer 2’s suggestion of the Sapra 2014 paper as a resource for aiding in our discussion of low Pseudo R-squares, and their implications for our models. This request led us to a fruitful, deeper investigation of model fit, and a much more conservative presentation of our results. We summarize the key points, below.  Per Reviewer 2’s recommendations: we have added a brief discussion of the Pseudo R square statistic to our methods section drawing on Sapra (2014), and highlighted Pseudo R squares in our results section. More importantly, we expanded our treatment of model evaluation to include three additional measures of goodness of fit: Pearson and Deviance chi square statistics, and dependent variable classification accuracy. Based on Pearson and Deviance chi squares, we removed Age as an independent variable from all models; this improved goodness of fit in all cases as well as decreasing the proportion of cells with counts of zero, strengthening all models.  Based on classification accuracy, we have limited our interpretation of our models to the largest levels of our dependent variables, consistent with the limited literature on multinomial logistic regressions that deal with rare events in one or more dependent variable level (Petrucci 2009). Although we explored options for correcting rare events bias derived from binomial logistic regression (Kosmidis and Firth 2011, King and Lanche 2001), we found that an available R package to implement one such option did not classify the dependent variable better than our original regressions. We retained the latter based on their otherwise strong goodness of fit statistics. The only exception to this case is our model for Relaxing Socially, which accurately predicted two levels of the dependent variable. |
| “General comment: Consider adding sub-headers in this section. The content and organization of the discussion section is really well-done, but sub-headers would aid the reader.” | We have added sub-headings to the discussion. |
| “Line. 421. This is an excellent point! Really insightful and important. However, this paragraph is a little confusing to read. Consider expanding this paragraph to allow of more clarification of your points. Just want to make sure this finding and related discussion comes through clearly.” | We have moved this argument in the discussion to reflect updated findings from our revised models. We have made efforts to increase clarity, and to expand the point in relation to the trends on activity engagement among un-employed respondents that we now report on. We integrate Fish et al. (2016)’s research on the interconnectedness of a wide range of activities and values from nature to support this point. |
| “Line 458. Can you provide an alternative estimate in your results section? More discussion is needed here, as mentioned above.” | As described above, we expanded our treatment of model evaluation to include three other measures of goodness of fit: Pearson and Deviance chi square statistics, and dependent variable classification accuracy |
| “Line 460. So should it be stated that these results should not be generalized outside of Vermont (or the region)?” | We have added a clarification that our results are likely indicative of predominantly rural, White areas of the country, but should be generalized with caution in other contexts. |
| “Line 462. What common biases arise from convenience sampling? Discuss and cite.” | We have expanded this discussion, added additional citations (Baker et al. 2013; Elliot & Haviland 2007), and noted that we included a financial incentive for survey participation to dull some of these biases. |
| **Journal Comments** | |
| “1. Please ensure that your manuscript meets PLOS ONE's style requirements, including those for file naming.” | We have included corresponding author information on the title page, and updated file names to match PLOS ONE’s style requirements. |
| “2. Please include additional information regarding the survey or questionnaire used in the study and ensure that you have provided sufficient details that others could replicate the analyses. For instance, if you developed a questionnaire as part of this study and it is not under a copyright more restrictive than CC-BY, please include a copy as Supporting Information.” | We have included a PDF of the survey instrument, exported from the lime survey platform, in our supplemental materials. |
| “3. In your Methods section, please provide additional information about the participant recruitment method and the demographic details of your participants” [a) recruitment date range (month, year), b) inclusion/exclusion criteria applied to participant recruitment, c) a table of relevant demographic details, d) statement of whether sample can be considered representative of a larger population, e) description of how participants were recruited, f) descriptions of where participants were recruited and where the research took place.” | We have expanded our methods section to provide more detail on participant recruitment including: recruitment data range; inclusion/exclusion criteria; and our online participant recruitment approach. We refer to our detailed table of respondent demographics (Table 1), and added a comment in Methods preceding this table to guide readers to the Limitations section where we discuss sample representativeness in detail. We have included a statement on research location at the end of the Data Processing sub-section of Methods. |
| “4. Please provide additional details regarding participant consent. In the ethics statement in the Methods and online submission information, please ensure that you have specified what type you obtained (for instance, written or verbal, and if verbal, how it was documented and witnessed)… If the need for consent was waived by the ethics committee, please include this information.” | We have provided more detail on our IRB approval, and the waiver of documentation of consent that we received, in our Methods section. Our discussion of participant recruitment in the first paragraph of Methods provides further details on the consent process. |
