# Supplementary material for: COVID-19 and human-nature relationships: Vermonters’ activities in nature and associated nonmaterial values during the pandemic
Source: PLoS One. 2020 Dec 11;15(12):e0243697. doi: 10.1371/journal.pone.0243697 (PMC7732125; doi:10.1371/journal.pone.0243697)
Supplement: Attachment — Submitted filename: Responses to Reviewers.docx [file pone.0243697.s013.docx]

Dear Dr. Martin-Ortega,

My co-authors and I appreciate the that time you, and our reviewers, have taken to consider our revised manuscript. We are likewise grateful for the additional comments offered the representativeness of our sample and the generalizability of our findings.

To the two suggestions in this round of reviews: we are glad to further qualify the representativeness of our sample, and offer our rationale for not commenting on the political leanings data we gathered, at the present time. In the table below, we address these thoughtful comments in greater detail. For ease of interpretation, we have grouped the comments about income in our sample as one row of replies, and the comments about the political data we collected as a separate row.

We have greatly appreciated the opportunity to strengthen this manuscript based on your, and our reviewers’, comments. Insights from this process have also been valuable in the follow-up work that we are currently conducting.

We look forward to your impressions of our most current revisions, and as always welcome any clarifying questions.

Sincerely,

Joshua Morse

Tatiana Gladkikh

Diana Hackenburg

Rachelle Gould

**REVIEWER RESPONSE TABLE**

| **Reviewer 2 Comment s** | **Response** |
| --- | --- |
| You say that "[O]ur results are likely indicative of relatively rural, White areas of the country." However, it is quite apparent that your sample is not representative of the median income-level across rural America-or of even America at large… As per my comment on the original submission, I feel this language concerning the representativeness of your sample needs further qualification. Your sample is significantly wealthier than rural America at large… I recommend that the language concerning the generalizability of your findings be limited to above average income households in Vermont and the surrounding region. | We have highlighted the skew towards above-median income among our respondents: “Noteworthy in this respect are the… skew towards higher income ranges among our respondents” (ln 586-599). We have also re-organized the Limitations section of our Discussion to better draw attention to the ways in which our sample is not representative of the Vermont population. Finally, we have adopted more conservative language about generalization: “With these considerations in mind we recommend caution in generalizing our results beyond predominantly White, rural areas in the Northeastern U.S.” (ln 611-612). We note that our income analysis defines the median range ($50,000-$74,999) quite evenly around the figure Reviewer #2 referred to in the 2016 Census Blog they cited ($62,291). |
| Additionally, you collected information on your samples' political leanings, but did not include this information in your supplemental data file or in your discussion of your sample. With the polarization occurring around the pandemic and our related human behavior, this seems pertinent. | We have chosen not to include the political variables we collected in our analysis for this paper, or to highlight them in our supplemental materials, because political leanings reflect values that many be closely intertwined with environmental values. Thus, the interplay between political leaning and ecosystem services/cultural ecosystem services prioritization merits more dedicated examination than this paper can accommodate. This is a subject that we are actively investigating in manuscripts currently in preparation that draw on this, and our follow-up, survey. |
