# Supplementary material for: COVID-19 and human-nature relationships: Vermonters’ activities in nature and associated nonmaterial values during the pandemic
Source: PLoS One. 2020 Dec 11;15(12):e0243697. doi: 10.1371/journal.pone.0243697 (PMC7732125; doi:10.1371/journal.pone.0243697)
Supplement: Attachment — Submitted filename: Reviewer Comments - Copy.docx [file pone.0243697.s011.docx]

**General Comments**

This is a timely, well-crafted manuscript that highlights a well-designed study concerning CES and COVID-19. Most of my comments represent minor revisions. The one exception being the need to reconsider how those values that did not fall into either factor are used in analysis. I feel this requires a revised analysis. If this is not undertaken and significant citation, rationale, and discussion is provided instead, it should be listed as a limitation, at a minimum.

Overall, however, I feel this provides valuable insight and clearly represents a commendable, rigorous study.

**Introduction**

Overall comment. Quite a bit of recreation research related to activity and COVID-19 has been conducted to date. Some of this is cited, but additional discussion of this research base is merited in the introduction.

Examples.

Venter, Z. S., Barton, D. N., Gundersen, V., & Figari, H. (2020). *Urban nature in a time of crisis: recreational use of green space increases during the COVID-19 outbreak in Oslo , Norway*. https://doi.org/https://doi.org/10.31235/osf.io/kbdum

Derks, J., Giessen, L., & Winkel, G. (2020). COVID-19-induced visitor boom reveals the importance of forests as critical infrastructure. *Forest Policy and Economics*, *118*, 102253.

O’Connell, T. S., Howard, R. A., & Hutson, G. (2020). The Impact of COVID-19 on Outdoor Recreation Participation in Canada.

Line 40. You may want to look to the work done following Hurricane Katrina for some insight in this area. It could be argued that we do know quite about how these large shocks impact the benefits we gain, values we place on nature, or perceive to gain from the natural world. You cite Rojas et al. (2017), but there is a larger body of work out there that directly speaks to this area you claim to be lacking.

Example:

Rung, A. L., Broyles, S. T., Mowen, A. J., Gustat, J., & Sothern, M. S. (2011). Escaping to and being active in neighbourhood parks: Park use in a post-disaster setting. *Disasters*, *35*(2), 383–403. https://doi.org/10.1111/j.1467-7717.2010.01217.x

Lines 47-63. Given the scope of this paper, the authors should be more specific in their discussion of ecosystem services, namely, specifying that many of these values/benefits are recreational ecosystem services, a sub-class of CES. Rice et al. (2020) discuss the need for conceptualizing and measuring non-material benefits of recreation at length in their discussion of recreational ES. This distinction is needed as RES are unique in the way they are attained, through direct, active interaction with the natural world. Therefore, they can actually be more easily measured than some other forms of CES which can provision their benefits more passively. Additionally, as noted by Rice et al., study of RES is also important as it brings intangible recreation benefits into a common lexicon of ES—thus legitimizing the importance of these benefits to decision-makers.

Rice, W. L., Newman, P., Taff, B. D., Zipp, K. Y., & Miller, Z. D. (2020). Beyond benefits: Towards a recreational ecosystem services interpretive framework. *Landscape Research*. https://doi.org/10.1080/01426397.2020.1777956

Line 81. For COVID-19 specific findings concerning urban-rural gradients see:
Rice, W. L., Mateer, T. J., Reigner, N., Newman, P., Lawhon, B., & Taff, B. D. (2020). Changes in recreational behaviors of outdoor enthusiasts during the COVID-19 pandemic: Analysis across urban and rural communities. *Journal of Urban Ecology*, *6*(1), 1–7. https://doi.org/10.1093/jue/juaa020

**Materials & Methods**

Line 107. How many other listservs and social media platforms were used in the data collection?

Line 113. What organization?

Lines 110-115. I would strongly encourage the authors to move this paragraph to the results section.

Line 120. How were these 15 activities selected? From previous research? Or the OIA or other list?

Lines 164-166. A citation is needed here.

Lines 188-189. Citation needed.

Line 202. A little confused here. Define “any combination”. Does this mean that if a participant designated one of the Factor 1 values as more important, they would be coded as 1 in the factor 1 dependent variable?

Line 202. I am similarly confused as to why values that were not included in either factor are included in both analyses: *“we classified participants who ranked any combination of factor 1 values and* ***values unassigned to either factor*** *as more important during the COVID-19 restrictions as “1””*. Language at the start of the paragraph does not suggest this. Additionally, it is unclear to the reader how many of those coded as “1” in the factor 2 dependent variable did not actually rank factor 2 values in their top three, but instead ranked unassigned values in their top three. Looking at Figure 2, the latter groups seems larger than the former. This has serious implications in your discussion of these results, and causes question towards the validity of this study. I understand that these unassigned values “loaded strongly with both factors,” but their inclusion in BOTH factors is not warranted and seems to undermine the validity of the study.

**Results**

Great job with the results section! I really like how the tables are displayed.

Line 238. These Pseudo R-squares are markedly low. I would recommend 1) discussing this statistic in the methods section (consider looking at Sapra (2014)), 2) being forthright about the low R^2^ in the results section, and 3) increasing the discussion of the limitations of this statistic and how these low R^2^s might impact the gravity of your findings in the limitations section.

Sapra, R. L. (2014). Using R^2^ with caution. *Current Medicine Research and Practice*, *4*(3), 130–134. https://doi.org/10.1016/j.cmrp.2014.06.002

**Discussion**

General comment: Consider adding subheaders in this section. The content and organization of the discussion section is really well-done, but subheaders would aid the reader.

Line. 421. This is an excellent point! Really insightful and important. However, this paragraph is a little confusing to read. Consider expanding this paragraph to allow of more clarification of your points. Just want to make sure this finding and related discussion comes through clearly.

Line 458. Can you provide an alternative estimate in your results section? More discussion is needed here, as mentioned above.

Line 460. So should it be stated that these results should not be generalized outside of Vermont (or the region)?

Line 462. What common biases arise from convenience sampling? Discuss and cite.
